# *Proteus mirabilis* and *Klebsiella pneumoniae* as pathogens capable of causing co-infections and exhibiting similarities in their virulence factors

**DOI:** 10.3389/fcimb.2022.991657

**Published:** 2022-10-20

**Authors:** Agata Palusiak

**Affiliations:** Laboratory of General Microbiology, Department of Biology of Bacteria, Institute of Microbiology, Biotechnology and Immunology, University of Łódź, Łódź, Poland

**Keywords:** core oligosaccharide, *Klebsiella pneumoniae*, lipopolysaccharide, *Proteus mirabilis*, virulence factors

## Abstract

The genera *Klebsiella* and *Proteus* were independently described in 1885. These Gram-negative rods colonize the human intestinal tract regarded as the main reservoir of these opportunistic pathogens. In favorable conditions they cause infections, often hospital-acquired ones. The activity of *K. pneumoniae* and *P. mirabilis*, the leading pathogens within each genus, results in infections of the urinary (UTIs) and respiratory tracts, wounds, bacteremia, affecting mainly immunocompromised patients. *P. mirabilis* and *K. pneumoniae* cause polymicrobial UTIs, which are often persistent due to the catheter biofilm formation or increasing resistance of the bacteria to antibiotics. In this situation a need arises to find the antigens with features common to both species. Among many virulence factors produced by both pathogens urease shows some structural similarities but the biggest similarities have been observed in lipids A and the core regions of lipopolysaccharides (LPSs). Both species produce capsular polysaccharides (CPSs) but only in *K. pneumoniae* these antigens play a crucial role in the serological classification scheme, which in *Proteus* spp. is based on the structural and serological diversity of LPS O-polysaccharides (OPSs). Structural and serological similarities observed for *Klebsiella* spp. and *Proteus* spp. polysaccharides are important in the search for the cross-reacting vaccine antigens.

## 1 Introduction

Both *Klebsiella* spp. and *Proteus* spp. are Gram-negative rods belonging to the *Enterobacterales* ord. nov (Brisse et al., 2006; Adeolu et al., 2016). In contrast to *Klebsiella* spp., *Proteus* spp. rods are motile (peritrichously flagellated) and capable of swarming. Swarming growth is triggered by a contact of short rod shaped swimmer cells (normally appearing in liquid medium) with 1.5% agar medium when the swimmer cells differentiate into elongated, hyperflagellated, multinucleated swarm cells. Swarmer cells move together across a plate till the population is reduced in its density and the consolidation process starts. In consolidation, which is regarded as a resting stage, swarmer cells re-differentiate into swimmer cells. The whole process is repeated till the characteristic bull’s-eye pattern appears on agar media (Armbruster and Mobley, 2012; Różalski et al., 2012; Schaffer and Pearson, 2015). By contrast, a feature, which distinguishes encapsulated *Klebsiella* spp. from *Proteus* spp. is a mucoid character of its colonies on agar media (Brisse et al., 2006). Both genera were described and named in the same year, 1885, *Klebsiella* – by Trevisan to honor the German microbiologist Edwin Klebs, and *Proteus* – by Hauser with reference to the Greek deity and the swarming nature of the bacteria (O’Hara et al., 2000; Brisse et al., 2006).

The taxonomy of each genus has been modified, although the taxonomic history of *Klebsiella* spp. aroused some controversial opinions concerning: the exclusion of *K. planticola* from the genus *Klebsiella* and transferring this species, together with *K. terrigena* and *K. ornithinolytica*, to the new genus *Raoultella* on the basis of the *rpoB* phylogenies (Podschun and Ullmann, 1998; Drancourt et al., 2001; Brisse et al., 2006). Currently, in the genus *Klebsiella* two major species are well known as causing infections in humans: *Klebsiella pneumoniae* with three subspecies – *pneumoniae*, *ozaenae* and *rhinoscleromatis* as well as *K. oxytoca*. Both species have been found to be genetically heterogeneous and comprise phylogenetic groups. *K. pneumonia* is subdivided into seven phylogroups (Kp1-Kp7) and *K. oxytoca* – into six phylogroups (Ko1-Ko4, Ko6, Ko8) corresponding to the following taxa: Kp1 – *K. pneumoniae*, Kp2 – *K*. *quasipneumoniae* subsp. *quasipneumoniae*, Kp3 – *K*. *variicola* subsp. *variicola*, Kp4 – *K. quasipneumoniae* subsp. *similipneumoniae*, Kp5 – *K*. *variicola* subsp. *tropicalensis*, Kp6 – *K*. *quasivariicola*, Kp7 – *K*. *africanensis*; Ko1 – *K*. *michiganensis*, Ko2 – *K*. *oxytoca*, Ko3 – *K*. *spallanzanii*, Ko4 – *K*. *pasteurii*, Ko6 – *K*. *grimontii* and Ko8 – *K*. *hauxiensis* (Gómez et al., 2021).

Phylogenetic analysis resulted in placing *Proteus* within a new *Morganellaceae* family (Adeolu et al., 2016). The genus *Proteus* includes three unnamed *Proteus* genomospecies 4, 5 and 6, ten named species: *P. mirabilis*, *P. vulgaris*, *P. penneri*, *P. hauseri* and six newly formed species, *P. cibarius*, *P. terrae*, *P. cibi*, *P. columbae*, *P. alimentorum* and *P. faecis* (Dai et al., 2019).

## 2 *P. mirabilis* and *K. pneumoniae* as etiologic agents of infections in humans

The representatives of both genera colonize the lower human intestinal tract, but *Klebsiella* spp. were more prevalent (Drzewiecka, 2016). *Klebsiella* spp. also colonize the nasopharynx (Brisse et al., 2006; Paczosa and Mecsas, 2016). The human digestive tract is regarded as a reservoir of both opportunistic pathogens leading to autoinfection or person-to-person transmitted nosocomial infections (Drzewiecka, 2016; Paczosa and Mecsas, 2016; Gómez et al., 2021). *Klebsiella* spp. accounted for 3-9.9% of all nosocomial bacterial infections and *Proteus* spp. (*P. mirabilis*) – for 3-5% of such infections depending on the survey (Brisse et al., 2006; Magill et al., 2014; Martin and Bachman, 2018). Among *Proteus* species, *P. mirabilis* is the most important etiological factor of infections (80-90% of all *Proteus* infections), which has also been confirmed by the studies performed on 617 *Proteus* spp. strains collected in Łódź (2006–2011) from different clinical sources (86.9% of isolation frequency) (Drzewiecka et al., 2021). Among *Klebsiella* species, *K. pneumoniae* is responsible for 75-86% of *Klebsiella* species infections (Watanakunakorn, 1991; Hansen et al., 2004). *K*. *pneumoniae* and *P. mirabilis* nosocomial infections include infections of the urinary tract (UTIs), wounds and bacteremia, which affect mainly immunocompromised patients especially in neonatal intensive-care units (*K. pneumoniae*) (Endimiani et al., 2005; Brisse et al., 2006; Różalski et al., 2012; Martin and Bachman, 2018; Gómez et al., 2021). On the contrary, a hypervirulent variant of *K. pneumoniae* (hvKP) is capable of causing serious infections *e.g.* endophthalmitis and meningitis, which can be metastatically spread to distant sites in non-immunocompromised ambulatory younger healthy hosts (Shon et al., 2013).

Both *K. pneumoniae* and *P. mirabilis* strains are etiologic agents of bloodstream infections (BSIs), where *K. pneumoniae* isolates which are regarded as one of predominant species among *Enterobacterales* bacilli (4.4-36% depending on the surveillance) cause BSI (Endimiani et al., 2005; Musicha et al., 2017; Leal et al., 2019; Kallel et al., 2020). *K. pneumoniae* BSIs often associated with the MDR phenotype. In contrast to the well-proven contribution of *K. pneumoniae* to BSIs, *P. mirabilis* are isolated less often (0.3-2.9%) from blood samples (Musicha et al., 2017; Leal et al., 2019; Kallel et al., 2020). An occurrence frequency of BSIs may also depend on a world region *e*.*g*. no *P. mirabilis* strains out of 536 collected in Łódź (Poland) came from blood (Drzewiecka et al., 2021). *P. mirabilis* BSIs mostly originate from complicated UTIs in patients with community-acquired infections (Chen et al., 2012). Two cases of *P. mirabilis* bacteraemia originating from infected aneurysm have also been noted (Nagiah and Badbess, 2018). *Klebsiella* bacteraemia (both primary and secondary) arise from a primary infection in the bladder or lungs (Paczosa and Mecsas, 2016).

Although *K. pneumoniae* is a primary cause of hospital-acquired pneumonia -11.8% of HAPs- (often severe with characteristic brick-red sputum and often leading to local necrosis in lungs), hospital-acquired pneumonia caused by *P. mirabilis* has also been reported (usually mild or moderate with a good efficacy rate, often affecting elderly patients with underlying diseases) (Wu and Li, 2015; Sękowska and Gospodarek, 2007; Okimoto et al., 2010; Magill et al., 2014; Paczosa and Mecsas, 2016). Both species have been relatively rarely found among pathogens causing community-acquired pneumonia (CAP) in Europe (Ko et al., 2002; Okimoto et al., 2010; Lin et al., 2010). However, *K. pneumonia* has been found to be the underlying agent of CAPs in Asia and Africa, which is related to the increased prevalence of hvKP in these regions (Ko et al., 2002; Paczosa and Mecsas, 2016).

*P. mirabilis* and *K. pneumoniae* species have also been reported to play a role in diarrheal disease (Lu. et al., 2017; Zhang et al., 2018; Gong et al., 2019). In China the detection rate of *K. pneumoniae* in faeces of persons with diarrhea was found to range from 0.5% among outpatients to 7.8% among hospital patients. In the majority of cases hypermucoviscosity phenotypes of *K. pneumoniae* were detected (Lu. et al., 2017; Zhang et al., 2018). Recently a novel diarrheagenic *P. mirabilis* strain (C02011) has been isolated in feces specimens in a food poisoning case in China. That strain was found to be a stronger gastrointenstinal pathogen than *P. mirabilis* B02005 isolated from healthy people. Moreover, the type IV secretion system (T4SS), also found in *K. pneumoniae*, was suggested to be crucial in the pathogenesis of diarrheal *P. mirabilis* (Gong et al., 2019). It is worth noting that both pathogens cause diarrhea in humans less often than other infections *e*.*g*. UTIs (O’Hara et al., 2000; Brisse et al., 2006).

*K. pneumoniae* and *P. mirabilis* cause community- and hospital-acquired urinary tract infections (UTIs) (Sękowska and Gospodarek, 2007; Cuevas et al., 2010; Chen et al., 2012). *K. pneumonia* is a causative agent of 2 to 6% of nosocomial UTIs and 4.3 to 7% of community-acquired ones (Laupland et al., 2007; Linhares et al., 2013; Paczosa and Mecsas, 2016). Depending on the studies and the world region, *P. mirabilis* placed third (behind *E. coli* and *Klebsiella* spp.) or fourth as a causative agent of UTIs (Coban et al., 2014; Tajbakhsh et al., 2015; Drzewiecka and Lewandowska, 2016). The isolation frequency from UTIs cases within one study was at a similar level for both pathogens and the numbers were much lower than those for *E. coli* (*E. coli* 81.8%, *K. pneumoniae* 7.9%, *P. mirabilis* 5.2% - Spain, 2008-2009) (Cuevas et al., 2010; Drzewiecka and Lewandowska, 2016). Both *K. pneumoniae* and *P. mirabilis* cause complicated UTIs in individuals with structurally abnormal urinary tracts, those undergoing long-term catheterization or patients with type 2 diabetes mellitus, but the latter species dominates as a causative agent of complicated UTIs (Dhingra, 2008; Drzewiecka and Lewandowska, 2016).

*P. mirabilis* and *K. pneumoniae* are a common cause of catheter-associated UTIs (CAUTIs) (Schroll et al., 2010; Armbruster et al., 2018). *P. mirabilis* CAUTIs often affect long-term catheterized patients (28 days or longer) where they lead to pyelonephritis, urolithiasis, prostatitis, stone and biofilm formation (Fox-Moon and Shirtliff, 2015; Armbruster and Mobley, 2012; Armbruster et al., 2018). In catheter biofilms *P. mirabilis* was found together with *K. pneumoniae*, representatives of both species were isolated more often from mixed-species biofilms (34.2% and 23.7%, respectively) than from single-species ones (20.0% and 3.3%, respectively), however this correlation can be better noticed for *K. pneumoniae* isolates (Macleod and Stickler, 2007). After the infection of 72 h *K. pneumoniae* biofilm by *P. mirabilis*, *K. pneumoniae* showed temporary antagonism against *P. mirabilis* and extended twice its mean time to catheter blockage compared to the control (Macleod and Stickler, 2007). Conversely, co-inoculation of *K*. *p*.-*P*. *m*. did not impact the *P. mirabilis* ability to attach to the siliconized surface but impaired the ability of *K. pneumonia* to bind to that surface (Galván et al., 2016). The persistence of UTIs caused by both pathogens may be connected with their increasing resistance to antibiotics, especially to nitrofurantoin commonly used in UTIs treatment. Moreover, the persistence of *P. mirabilis*-associated UTIs may result from urolithiasis that may lead to the catheters and urinary tract obstruction (Jacobsen et al., 2008; Cuevas et al., 2010; Drzewiecka and Lewandowska, 2016). 65% of specimens from patients with long-term catheterization contained *P. mirabilis*, which was associated with the catheter obstruction (Mobley and Warren, 1987). The dual-species associations of *K*. *p*. with *P*. *m*. were found to account for 7% of the 97% polymicrobial cases of CAUTIs in a two-year survey (Galván et al., 2016).

Apart from polymicrobial UTIs, other infections may also be caused by the representatives of both species *e.g. P. mirabilis* and *K. pneumoniae* were isolated from the abdominal wound of a man with the carcinoma of the colon (Krajden et al., 1987). What is interesting, both species have been implicated in chronic inflammatory arthritis: *K. pneumoniae* in ankylosing spondylitis (AS) and *P. mirabilis* in rheumatoid arthritis (RA). In both cases the role of *K. pneumoniae* and *P. mirabilis* in the development of AS or AR, respectively, was associated with the molecular similarity between amino acid sequences of bacterial antigens and appropriate human self-antigens (**Table 1**) (Brisse et al., 2006; Rashid and Ebringer, 2007a; Rashid and Ebringer, 2007b; Różalski et al., 2012). The etiopathogenic role of *K. pneumoniae* in the AS development is connected with a molecular homology between: 1) ‘QTDRED’ amino acid sequences present in both *K. pneumoniae* nitrogenase reductase enzyme and HLA-B27, 2) between ‘DRDE’ amino acid sequences found in PulD secretion proteins of *K. pneumoniae* pullulanase enzyme and the ‘DRED’ sequence in HLA-B27, 3) between repeating sequences occurring in PulA of *Klebsiella* spp. pullulanase and types I, III and IV collagens (Schwimmbeck et al., 1987; Fielder et al., 1995). On the other hand, the potential role of *P. mirabilis* in the etiopathogenesis of RA is associated with molecular similarity between: 1) the ‘ESRRAL’ amino acid sequences found in *P. mirabilis* hemolysins and the ‘EQ/KRRAA, motif observed in RA-associated HLA-DR molecules, 2) between the ‘LRREI’ sequences in *Proteus* urease and the ‘IRRET’ motif present in type XI collagen. In both cases high titers of antibodies against *K. pneumoniae* or *P. mirabilis* were detected in the sera of the patients with active AS or RA, respectively (Ebringer et al., 1992; Wilson et al., 1995; Ebringer et al., 2003; Ebringer et al., 2006). As a consequence of the mimicry phenomenon, bacterial antigen-specific antibodies, acting as autoantibodies, recognize also human self-antigens, which results in the damage to hyaline cartilage (Wilson et al., 1995; Konieczna et al., 2012). Different body sites are regarded as the isolation sources of both pathogens in RA or AS infections – the upper urinary tract of RA patients (*P. mirabilis*) and the colon of AS patients (*K. pneumoniae*) (Rashid and Ebringer, 2007a; Rashid and Ebringer, 2007b).

**Table 1:** Molecular similarities between human self-antigens and appropriate *P. mirabilis* and *K. pneumoniae* enzymes (Schwimmbeck et al., 1987; Ebringer et al., 1992; Fielder et al., 1995; Wilson et al., 1995; Ebringer et al., 2003; Ebringer et al., 2006; Rashid and Ebringer, 2007a; Rashid and Ebringer, 2007b; Konieczna et al., 2012).

*K. pneumoniae*		Human self-antigens	
Nitrogenase reductase	**Glu-Thr-Asp-Arg-Glu-Asp**	HLA-B27	**Glu-Thr-Asp-Arg-Glu-Asp**
Pullulanases:			
Pul-D	**Asp-Arg-**Asp-Glu	HLA-B27	Glu-Thr-**Asp-Arg**-Glu-Asp
Pul-A	**Gly-X-Pro**	Collagens type I, III and IV	**Gly-X-Pro**
*P. mirabilis*			
Hemolysin	Glu-Ser-**Arg-Arg-Ala**-Leu	HLA-DR1/4	Glu-Gln-**Arg-Arg-Ala**-Ala
Urease	Leu-**Arg-Arg-Glu**-Iso	Collagen type XI	Iso-**Arg-Arg-Glu**-Thr

*amino acids sequences common for the bacterial antigen and appropriate self-antigen are bolded.

## 3 The virulence factors with similar features observed for *P. mirabilis* and *K. pneumoniae* strains

*P. mirabilis* and *K. pneumoniae* along with *Escherichia coli* are the most common causes of UTI cases, both community- and hospital-acquired ones (Oloomi et al., 2020). The first two species contribute to recurrent UTIs, which derives from their multidrug-resistance (Hooton, 2001). These facts and co-isolation of both species from polymicrobial infections increase the interest in the similarities between virulence factors of *K. pneumoniae* and *P. mirabilis* (Macleod and Stickler, 2007; Galván et al., 2016). Many factors including: adhesins, siderophores, protein toxins, proteinases, ureases are implicated in the virulence of the representatives of both species, and a few have much in common in their structure and functions (Lindberg et al., 1975; Dutton et al., 1978; Joseleau et al., 1978; Joseleau and Marais, 1979; Sidorczyk et al., 1983; Joseleau and Marais, 1988; Jansson et al., 1994; Erbing et al., 1995; Mobley et al., 1995; Helander et al., 1996; Podschun and Ullmann, 1998; O’Hara et al., 2000; Vinogradov et al., 2002; Brisse et al., 2006; Holst, 2007; Knirel et al., 2011; Różalski et al., 2012; Konieczna et al., 2012; Kubler-Kielb et al., 2013). Urease, LPS and capsule polysaccharides will be discussed in detail in the next chapters as the virulence factors with the highest number of features common to both species (Lindberg et al., 1975; Dutton et al., 1978; Joseleau et al., 1978; Joseleau and Marais, 1979; Sidorczyk et al., 1983; Joseleau and Marais, 1988; Jansson et al., 1994; Erbing et al., 1995; Mobley et al., 1995; Helander et al., 1996; Vinogradov et al., 2002; Holst, 2007; Knirel et al., 2011; Konieczna et al., 2012; Kubler-Kielb et al., 2013).

### 3.1 Urease as the non-polysaccharide virulence factor with similar features observed for *P. mirabilis* and *K. pneumoniae* strains

Urease is the nickel containing enzyme catalyzing the hydrolysis of urea to yield ammonia and carbon dioxide. Ammonia has been found to damage the tissues and elevate urine pH resulting in crystallization of magnesium and calcium ions, which contributes to stone formation (Maroncle et al., 2006). In *Klebsiella* spp. urease is localized in cytoplasm and in *P. mirabilis* it is present in periplasm or in the outer membrane. *P. mirabilis* urease is induced by the presence of urea and *K. pneumoniae* urease – by the presence of poor nitrogen sources (Mobley et al., 1995).

Urease is a non-polysaccharide virulence factor, which shows structural similarities in both *Klebsiella* spp. and *Proteus* spp (Mobley et al., 1995; Stickler et al., 1998; Różalski et al., 2012). Similarities have been found in gene sequences in urease gene clusters (*ureDABCEFG*) in *K. aerogenes* and *P. mirabilis*. In contrast to *K. aerogenes* genome, in the *P. mirabilis* genome *ureR* gene (*ureR*-dependent promoter) was additionally detected - upstream of *ureD* (Mobley et al., 1995; Island and Mobley, 1995; Coker et al., 2000). The *ureR* regulates the urease expression in *Proteus* spp., and in *Klebsiella* spp. this role is played by the nitrogen regulatory system (NTR). Predicted molecular sizes of appropriate ure-encoded polypeptides are also similar in *K. aerogenes* [1] and in *P. mirabilis* [2]:

[1] UreA 11.1 kDa, UreB 11.7 kDa, UreC 60.3 kDa, UreE 17.6 kDa, UreF 25.2 kDa, UreG 21.9 kDa;
[2] UreA 11.0 kDa, UreB 12.2 kDa, UreC 61.0 kDa, UreE 17.9 kDa, UreF 23.0 kDa, UreG 22.4 kDa (Mobley et al., 1995).

The protein sequence of the UreC subunit from *K. aerogenes* is 72% identical to the UreC sequence from *P. mirabilis* (Mobley et al., 1995). Although urease is regarded as a conservative enzyme and its structure is similar for different bacteria, except *Helicobacter pylori* or *Staphylococcus cohnii*, the numbers of amino acids of particular polypeptides and the structure of the flap region in ureases of *P. mirabilis* and *K. aerogenes* show the highest homology. From the protein products of *ure* genes described for *P. mirabilis* and *K. pneumoniae* the highest similarities were observed for UreD [274 amino acids], UreG [205], α [567] and γ [100] (Konieczna et al., 2012).

More divergences noted for *P. mirabilis* and *K. pneumoniae* urease concerned its activity. The latter species has been reported to hydrolyze urea ten times slower than the former one (Stickler et al., 1998). Other studies using different solid media have also confirmed that correlation, i.e. the onset of urease reaction appeared after 9-10 h in the case of *Klebsiella* spp. strains and after 1-2 h for *Proteus* spp. (Vuye and Pijck, 1973). Some *Klebsiella* sp. strains are urease-negative, but they were found to enhance the *P. mirabilis* urease activity in the *K*. *p*. – *P*. *m*. coculture experiment (Armbruster et al., 2017). The underlying mechanism of enhanced urease activity of one strain by another may decrease the risk of development of complications in polymicrobial CAUTIs (Armbruster et al., 2017), thus elimination of one bacterial species may weaken the urease activity of the representatives of other species.

As was mentioned before, urease activity is connected with urinary stones occurrence. *Proteus* spp. is one of the most common pathogens inducing the formation of bladder and kidney stones, mainly struvite ones. Although *Klebsiella* spp. are mentioned as urease producing pathogens, also isolated from patients with urinary stones (mainly calculi containing oxalate and calcium phosphate) (Bichler et al., 2002; Torzewska et al., 2014), they have been found to produce none or little catheter encrustation and not to elevate urinary pH above 7.0 (Stickler et al., 1998; Broomfield et al., 2009). However, *K. pneumoniae* is known to produce mucoid plugs in the catheter lumen (Broomfield et al., 2009). *P. mirabilis* have been demonstrated to cause crystallization within the human ureter and bladder epithelial cells (Torzewska et al., 2014). It has been shown that the crystallization process may be enhanced (*P. vulgaris* O12) or inhibited (*P. mirabilis* O28 and O47) by the *Proteus* lipopolysaccharides (LPSs) depending on their O-polysaccharides (OPSs) structures and their affinity for Ca^2+^ and Mg^2+^ cations (Torzewska et al., 2003). Despite the role of bacterial urease in UTIs development, the metabolism of urea has also been demonstrated to be important in the gastro intestinal tract colonization by *K. pneumoniae* (Maroncle et al., 2006).

Urease is also produced by other *Enterobacterales* which are a part of human intestinal microflora but the level of the enzyme activity is species-specific and varies with the medium used (Vuye and Pijck, 1973). For example, among *Escherichia coli* strains, only 1% have been identified as urease-positive (Konieczna et al., 2012). *Citrobacter* and *Enterobacter* spp. are characterized as those with irregular urease activity. Among urease-positive *Serratia* spp. strains, urease action is delayed more than in the case of *Klebsiella* spp. strains (Vuye and Pijck, 1973). Despite the fact that *Providencia morganii* and *P. rettgeri* are regarded to be as effective urease producers as *P. mirabilis*, the urease activity, structure and genetic organization of the enzyme genes are well-known for *Klebsiella* and *Proteus* spp. frequently isolated from polymicrobial UTIs (Mobley et al., 1995; Konieczna et al., 2012; Galván et al., 2016).

### 3.2 Polysaccharide virulence factors with similar features observed for *P. mirabilis* and *K. pneumoniae* strains

#### 3.2.1 Lipopolysaccharide

LPS is an essential component of the outer membrane of all Gram-negative bacteria and their major virulence factor. Due to its biological activity, LPS is regarded as an endotoxin. During infection it is released from bacterial cells to the blood where it is bound to LPS binding protein (LBP) and through CD14 and TLR4 (Toll-like receptor) induces macrophages to the pro-inflammatory cytokines secretion. Over-stimulation of the process leads to endotoxic shock development (Poxton, 1995). LPS exhibits many biological activities and its parts interact with bacterial or eukaryotic cells, *e.g*. O-polysaccharides chains, most outstanding from the bacterial cell, are involved in glycocalyx formation or make the bacteria resistant to the active membrane attack complex (Poxton, 1995; Różalski et al., 2012). It is worth mentioning that LPS is highly immunogenic and its parts induce the production of antibodies of different specificity, which is important in searching for broadly cross-reacting immunoglobulins (Poxton, 1995).

Smooth (S) forms of *Proteus* spp. and *Klebsiella* spp. bacteria produce LPS consisting of three parts differing in their chemical structures and biological functions: lipid A, a core region and an OPS (Vinogradov et al., 2002; Palusiak, 2013).

##### 3.2.1.1 Lipid A

Lipid A anchors LPS in the outer leaflet of the bacterial outer membrane (Poxton, 1995). Lipid A is structurally the most conserved region of LPS among Gram-negative bacteria and in *P. mirabilis* and *K. pneumoniae* bacilli the lipid A structures are almost identical (Sidorczyk et al., 1983; Helander et al., 1996).

The lipid A structure of *Proteus* spp. LPS has been established for the deep rough R45 mutant of the *P. mirabilis* S1959 strain (Sidorczyk et al., 1983). The lipid A structure of *Klebsiella* spp. LPS was characterized in 1996 for the polymyxin-resistant *K. pneumoniae* O3 mutant OM-5 and its polymyxin-sensitive parent strain (LEN-1) (Helander et al., 1996). *P. mirabilis* R45, *K. pneumoniae* LEN-1 and OM-5 lipids A are similar in the fatty acids arrangement and composition. All contain seven fatty acids residues (heptaacylated lipid A) including four 3-hydroxytetradecanoic acids (3-OH-C_14_) attached directly to the backbone (bisphosporylated β(1→6) linked GlcN disaccharide), two tetradecanoic acids ester-linked to 3-OH-C_14_ linked to the nonreducing GlcN residue and one hexadecanoic acid that partially substitutes the hydroxyl group of 3-OH-C_14_ acid present at position 2 of reducing GlcN (Kotełko, 1986; Helander et al., 1996). For *K*. *pneumoniae* and *P*. *mirabilis* the hexaacylated species of lipids A (without hexadecanoic acid) have also been observed. In lipid A of LPSs from both species the ester-bound phosphate residue is substituted by 4-amino-4-deoxy-L-arabinopyranose (L-Ara*p*4N). The feature, which probably distinguishes *P. mirabilis* R45 lipid A of LPS from lipids A of *K. pneumoniae* LEN-1 and OM-5 LPSs is a free glycosidically bound phosphate group, which in *K. pneumoniae* lipid A is substituted by L-Ara*p*4N (Kotełko, 1986; Helander et al., 1996). However, L-Ara*p*4N may be bound to the glycosidic phosphate group in a labile diester linkage, which could be cleaved under strong alkaline conditions. Thus, the structural analysis of *P. mirabilis* R45 LPS indicates only the putative substitution of a glycosidic phosphate residue by a pentosamine residue (Vinogradov et al., 1994). In *K. pneumoniae* LEN-1 and OM-5, the L-Ara*p*4N is linked to both phosphates of the lipid A backbone. However, the degree of substitution of phosphates by L-Ara*p*4N in both LPSs is remarkably different (Helander et al., 1996).

Lipid A of *Proteus* spp. as well as of other *Enterobacterales* ord. nov. is linked to the core region by a hydroxyl group at C6’ of a nonreducing GlcN residue (Kotełko, 1986).

##### 3.1.1.2 The core oligosaccharide of LPS

**Figure 1:**
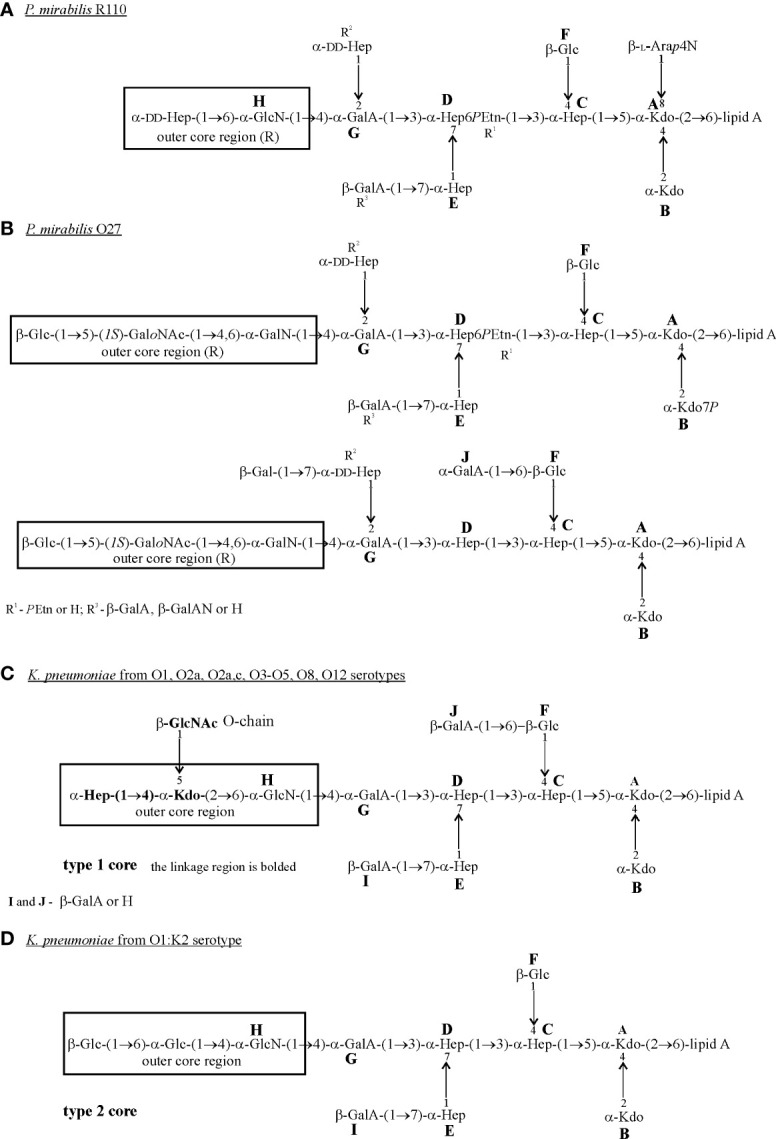
Structures of the core regions of: **(A)** *P. mirabilis* R110; **(B)** *P. mirabilis* O27; **(C)** *K. pneumoniae* from O1, O2a, O2a,c, O3-O5, O8 and O12 serotypes; **(D)** *K*. *pneumoniae* from the O1:K2 serotype (Vinogradov et al., 2000; Vinogradov and Perry, 2001; Vinogradov et al., 2002; Regué et al., 2005; Holst, 2007). The outer core region **(R)** is indicated by the frame. Ara*p*4N, 4-amino-4-deoxy-L-arabinopyranose; GalA, galacturonic acid; GalAN, GalA amidated by aliphatic polyamines; GalN, galctosamine; Gal*o*NAc, open-chain of GalNAc; Glc, glucose; GlcN, glucosamine; GlcNAc, 2-acetamido-2-deoxy-D-galactose; LD-Hep, L-*glycero*-D-*manno*-heptose; DD-Hep, D-*glycero*-D-*manno*-heptose; Kdo, 3-deoxy-D-*manno*-oct-2-ulosonic acid; *P*Etn,2-aminoethyl phosphate.

Comparison of the structures of the LPS core regions in all tested *Proteus* spp. and *K. pneumoniae* strains shows that they share a common heptasaccharide fragment, which includes:

➢ a disaccharide of 3-deoxy-α-D-*manno*-oct-2-ulosonic acid (α-Kdo; A, B);
➢ a trisaccharide of L-*glycero*-α-D-*manno*-heptose (α-Hep; C, D, E);
➢ β-glucose (β-Glc; F) residue linked to α-Hep C at O (4);
➢ an α-galacturonic acid (α-GalA; G) residue linked to α-Hep D at O (3) (**Figure 1**) (Vinogradov et al., 2000; Vinogradov and Perry, 2001; Vinogradov et al., 2002; Regué et al., 2005; Holst, 2007; Vinogradov, 2011).

This heptasaccharide fragment is also a part of the LPS core region of two *Serratia marcescens* strains (Holst, 2007; Palusiak, 2013).

Other common residues which can be found in all tested LPS core regions of *K. pneumoniae* and some *P. mirabilis* strains, are: α-GlcN (H) attached to the α-GalA (G) residue at O4 and β-GalA (I/R^3^) linked to Hep (E) at O (7) (**Figure 1**) (Vinogradov et al., 2000; Vinogradov and Perry, 2001; Vinogradov et al., 2002; Regué et al., 2005; Holst, 2007; Vinogradov, 2011). A component common for *P. mirabilis* (O27) (**Figure 1B**) and *K. pneumoniae* (O1, O2a, O2a,c, O3-O5, O8, O12) LPS core regions (**Figure 1C**) is GalA (J) substituting β-Glc (F) at O (6) (non-stoichiometric substitution) (Vinogradov and Perry, 2001; Vinogradov et al., 2002; Holst, 2007). Major structural differences between LPS core regions of *P. mirabilis* and *K. pneumoniae* strains concern the outer core region (**Figure 1**) (Vinogradov et al., 2002; Regué et al., 2005).

It is worth mentioning that over 21 different structures of the outer core region have been determined in *Proteus* spp., among which eight have been characterized for *P. mirabilis* strains. These structures are mainly linear or branched (*P. mirabilis* O48) and contain from two to four sugar residues, some of which are acylated (Vinogradov et al., 2002; Vinogradov, 2011). In *K. pneumoniae* only two structures of the LPS core region differing in the substituents of α-GlcN (H) have been described (**Figures 1C, D**) (Vinogradov and Perry, 2001; Regué et al., 2005). Neither LD-Hep nor α-Kdo, present in the outer core region of *K. pneumoniae* LPSs (type 1 core) (**Figure 1C**), has ever been found in this part of *Proteus* spp. LPSs (Vinogradov et al., 2002; Vinogradov, 2011). Also, the *P. mirabilis* LPS outer core region may contain components which are not found in the *K. pneumoniae* LPS core region *e.g.* cyclic acetal formed by an 2-acetamido-2-deoxy-D-galactose residue in the open-chain form (Gal*o*NAc) linked to O (4) and O (6) of the neighboring GalN residue (*P. mirabilis* O27) (**Figure 1B**) (Vinogradov et al., 2002).

The great structural heterogeneity of *Proteus* spp. LPS core regions does not result from the differences only in the outer core region but also in the inner core region, which is in general more structurally conserved in other *Enterobacterales* ord. nov. LPSs (Vinogradov et al., 2002; Palusiak, 2013).

In contrast to *K. pneumoniae* LPSs, three glycoforms differing in two substituents R^2^ (α-DD-Hep-(1→2)-α-DD-Hep, α-DD-Hep or H) and R^3^ (β-GalA or H) have been determined for the inner core region of *Proteus* spp. LPSs. The majority of *P. mirabilis* LPSs tested so far have presented glycoform II of their inner core region (**Figures 1A, B**) (Vinogradov et al., 2002; Kondakova et al., 2005). What is more interesting, multiple structural variants of the *P. mirabilis* core region may appear even within the LPS of one strain *e.g.* two types of the core region in *P. mirabilis* O27 (**Figure 1B**) and four different R^1^ (*P*Etn), R^2^, R^3^ combinations in the *P. mirabilis* O3 LPS core region (Vinogradov et al., 2002), which does not occur in the case of *K. pneumonia* (Holst, 2007).

The core region of the *P. mirabilis* O27 LPS is similar to the core region of *K. pneumoniae* LPS due to the lack of L-Ara*p*4N linked to the Kdo (A) residue at O (8) (**Figures 1B–D**) (Regué et al., 2005; Holst, 2007). L-Ara*p*4N occurs at this position in the other tested *Proteus* spp. LPSs (**Figure 1A**) (Vinogradov et al., 2002) and has been found to play a crucial role in the decreased binding of polymyxin B (Boll et al., 1994).

##### 3.1.1.3 O-polysaccharide of LPS

**Figure 2:**
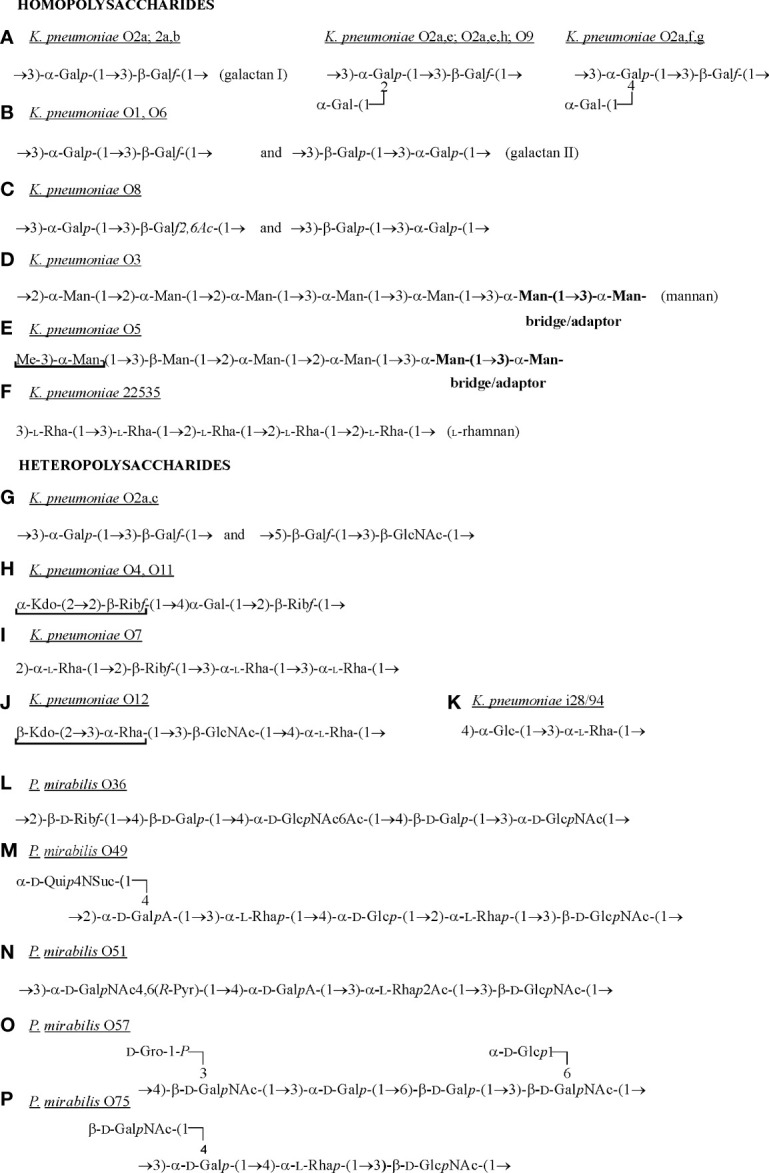
Structures of *K. pneumoniae* O-polysaccharides **(A–K)** and of those *P. mirabilis* OPSs **(L-P)**, which have the components common with the selected *K. pneumoniae* OPSs (Knirel, 2011; Knirel et al., 2011). The residues which do not belong to the repeating units are underlined. Gal*f*, galactofuranose; Gal*p*, galactopyranose; Glc, glucose; GlcNAc 2-acetamido-2-deoxy-D-galactose; GalA, galacturonic acid; D-GalNAc, 2-acetamido-2-deoxy-D-galactose; D-Gro-1-*P*, D-glycerol 1-phosphate; Kdo, 3-deoxy-D-*manno*-oct-2-ulosonic acid; Man, mannose; Pyr – pyruvic acid; Rha, ramnose; Rib*f*, ribofuranose; Suc, succinic acid; Qui4n, 4-amino-4-deoxy-D-quinovose.

To date, the O-antigen linkage in *Proteus* spp. LPS has not been precisely determined. However, the R substituent of the core region (**Figure 1**) is indicated as a possible linkage site of OPS (Vinogradov et al., 2002). The *K. pneumoniae* type 1 core oligosaccharide is linked to OPS *via* O-5 of the Kdo residue from the outer core region (**Figure 1C**) (Vinogradov et al., 2002; Holst, 2007). This Kdo residue is a part of the linkage region, which is common to all tested *K. pneumoniae* O antigens (**Figure 1C**), the bolded fragment. The primer β-GlcNAc residue forming the linkage region is derived from the O-chain biosynthesis pathway and is substituted by a repeating unit of the majority of *K. pneumoniae* O antigens or by the bridging disaccharide 3)-(α-Man-(1→3)-α-Man) in *K. pneumoniae* O3 and O5 OPSs (**Figures 2D, E**) (Vinogradov et al., 2002; Knirel, 2011). In *K. pneumoniae* strain 52145 from serotype O1:K2 (type 2 OS) (**Figure 1D**) the last Glc outer-core residue has been suggested to be a linkage site of OPS (Regué et al., 2005).

**Figure 3:**
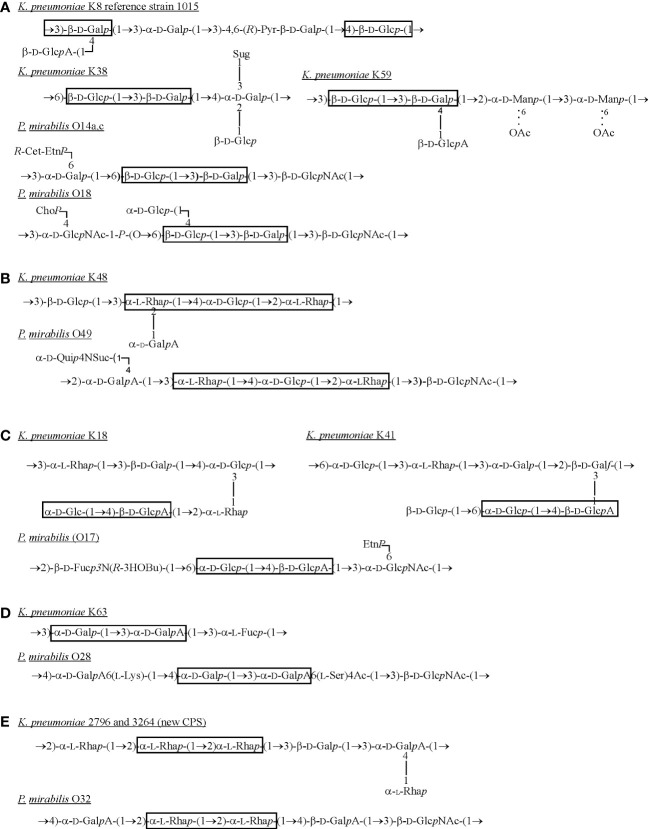
Structures of *K. pneumoniae* capsular polysaccharides and *P. mirabilis* OPSs **(A–E)** sharing the common fragments (in frames) (Lindberg et al., 1975; Dutton et al., 1978; Joseleau et al., 1978; Joseleau and Marais, 1979; Joseleau and Marais, 1988; Jansson et al., 1994; Erbing et al., 1995; Knirel et al., 2011; Kubler-Kielb et al., 2013). Dotted lines indicate that some of the residues are *O*-acetylated. Cho*P*, choline phosphate; Etn*P*, ethanolamine phosphate; Fuc, fucose; D-Fuc3N, 3-amino-3-deoxy-D-fucose; Gal*f*, galactofuranose; Gal*p*, galactopyranose; GalA, galacturonic acid; Glc, glucose; GlcA, glucuronic acid; D-GlcNAc, 2-acetamido-2-deoxy-D-glucose; L-Lys, L-lysine; Man, mannose; Pyr – pyruvic acid; *R*-3HOBu, (*R*)-3-hydroxybutanoic acid; *R*-Cet-Etn*P*, *N*-[(*R*)-1-carboxyethyl]ethanolamine phosphate; Rha, ramnose; L-Ser, L-serine; Suc, succinic acid; Sug, 4-deoxy-*threo*-hex-4-enopyranosyluronic acid group; Qui4N, 4-amino-4-deoxy-D-quinovose.

The OPS of *P. mirabilis* is definitely more structurally heterogenous than that of *K. pneumoniae*. For *P. mirabilis* strains, 49 different OPS structures have been determined and some of them (*e.g. P. mirabilis* O69 OPS) are common to other representatives of the genus (Knirel et al., 2011; Drzewiecka et al., 2016a; Drzewiecka et al., 2016b). The OPS structures of the most *Proteus* strains tested so far have been already gathered and presented in a review by Knirel et al. (2011) (Knirel et al., 2011). Therefore, in this review only structures of OPSs, which share common fragments with *Klebsiella* CPSs are presented (**Figure 3**).

In *K. pneumoniae* only 13 OPS structures have been determined (Knirel, 2011). Most OPS structures presented by *P. mirabilis* strains are branched, and in *K. pneumoniae* LPSs the lateral substituent appears only in two OPS structures (**Figure 2A**) (Knirel, 2011; Knirel et al., 2011). *P. mirabilis* OPSs consist of repeating units comprising from three to seven sugar and non-sugar components and *K. pneumoniae* repeating units include from two to five sugar components. *Proteus* spp. OPSs are heteropolysaccharides, whereas, among *K. pneumoniae* O-antigens, homopolysaccharides also occur (*e.g.* O3, O5, 22535) (**Figures 2D–F**). Homopolymers are also present in *K. pneumoniae* OPSs in a form of galactan I: 3)-α-Gal*p*-(1→3)-β-Gal*f*(1-, which forms the repeating units alone (e.g. O2a, O2a,b OPSs (**Figure 2A**) or together with galactan II: 3)-β-Gal*p*-(1→3)-α-Gal*p*(1- (O1, O6 and O8) (**Figures 2B, C**) (Knirel, 2011). The latter is placed distally toward galactan I and defines the O1 serotype (Vinogradov et al., 2002).

The β-Gal or α-Gal residues are found in *P. mirabilis* OPSs quite frequently but contrary to the β-Gal residues in *Klebsiella* OPS, they always occur in a pyranose form. These residues are present in *P. mirabilis* OPSs as single Gal residues (*P. mirabilis* O75) (**Figure 2P**) or as Gal disaccharides but in a different sequence than in galactan II in *K. pneumoniae* OPS *e.g.* 3)-α-Gal*p*-(1→6)-β-Gal*p*(1- (*P. mirabilis* O57) (**Figure 2O**) (Vinogradov et al., 2002; Knirel et al., 2011).

Unlike *P. mirabilis* OPSs, the OPS chains of *K. pneumoniae* O4, O5, O11 and O12 antigens possess at their non-reducing ends residues that are not composed of the repeating units *e.g.* α-Kdo-(2→2)-β-Rib*f* (O4), Me-3)α-Man (O5) or β-Kdo-(2→3)-α-Rha (O12) (**Figures 2E, H, J**) - underlined residues (Vinogradov et al., 2002). Kdo or Man residues have not been found in *P. mirabilis* OPSs so far (Knirel et al., 2011). Other repeating units structures of *K. pneumoniae* OPSs are heteropolymers (**Figures 2G–K**) (Vinogradov et al., 2002; Knirel, 2011). The single components of the *K. pneumoniae* O12 OPS (**Figure 2J**) are also found in *P. mirabilis* OPSs: α-Rha-(1→3)-β-D-GlcNAc(1- (*P. mirabilis* O49 and O75 or in O51, where α-Rha is 2-O-acetylated) (**Figures 2M, N, P**). Components similar to those found in the repeating units of the *K. pneumoniae* O4 and O11 antigens [4)-α-Gal-(1→2)-β-Rib*f*(1-] (**Figure 2H**) have also been detected in the *P. mirabilis* O36 antigen: 2)-β-Rib*f*-(1→4)-β-Gal(1- (**Figure 2L**) (Knirel et al., 2011; Knirel, 2011).

###### 3.1.1.3.1 *Klebsiella* spp. and *Proteus* spp. O-typing schemes

The O-antigen determines the serological specificity of the S and SR forms of bacteria (Knirel et al., 2011). The classification scheme for *Proteus* spp. was founded by Kauffmann (1966) and included 49 different *P. mirabilis* and/or *P. vulgaris* O serogroups (Kauffmann, 1966). This scheme was completed by Larsson et al. (1978) (six *P. mirabilis* O-serogroups) (Larsson et al., 1978), by Penner and Hennessy (1980) (11 O-serogroups containing *P. mirabilis* or *P. vulgaris* strains) (Penner and Hennessy, 1980) and by other authors (O-serogroups consisting of different *Proteus* species or of one species) (Knirel et al., 2011; Kaca et al., 2011; Palusiak, 2013; Palusiak et al., 2013). Currently, the *Proteus* O-antigen classification scheme includes 83 O-serogroups, among which 43 contain *P. mirabilis* strains (the majority of them contain *P. mirabilis* strains only and in eight O-serogroups there are also other representatives of the genus – O8, O11, O13, O17, O23, O34, O54, O69 and O71) (Knirel et al., 2011; Drzewiecka et al., 2016a; Drzewiecka et al., 2016b). O77-O82 serogroups were created from *Proteus* clinical isolates from the Łódź area (Central Poland) (Drzewiecka et al., 2021).

The serological classification of *Klebsiella* spp. strains was reported in 1940 by Kauffmann and Ørskov, who separated three O groups: 1, 2 (with 2A and 2B subgroups) and 3, among which the O1 group was the most numerous (Ørskov, 1954). Initially, 12 O-antigen serogroups were described but structural similarities and the cross-reactivities between some of them resulted in slight differences in the authors’ opinions on the *Klebsiella* O typing scheme (Trautmann et al., 1997; Hansen et al., 1999; Vinogradov et al., 2002). For example, Trautmann et al. (1997) described the *Klebsiella* O9 serogroup as a separate one but the investigation by Hansen et al. (1999) revealed that the LPS from the strain prototype for serogroup O9 was structurally and serologically identical to the LPS from the strain belonging to serogroup O2 (Trautmann et al., 1997; Hansen et al., 1999). Thus serogroup O9 was proposed to be treated as a part of serogroup O2 (Hansen et al., 1999). Trautmann suggested excluding serogroup O8 (together with O6) from the scheme since O8 antigens had been found to be serologically indistinguishable from O1 LPSs (Trautmann et al., 1997; Trautman et al., 2004). On the contrary, Hansen et al. (1999) presented serogroup O8 as a separate one, mentioning that genetic methods would help in the distinction between O1 and O8 representatives (Hansen et al., 1999).

Among *Klebsiella* spp. O serotypes, O1 (the most often), O2 and O5 dominated among clinical isolates from Japan, Germany, Denmark, Spain and the United States with the exception of blood isolates from the USA where O2 representatives dominated over O1 (Trautmann et al., 1997; Hansen et al., 1999; Brisse et al., 2006). As for *Proteus* spp., the O3 serogroup dominated among *P. mirabilis* isolates according to the studies described by Larsson. In most of the studies *Proteus* O10 and O30 serogroups were also frequently found (Larsson, 1984). The O78 serogroup was found to be the most numerous in the *Proteus* classification scheme and seemed to be prevalent among patients from the Łódź area (Drzewiecka et al., 2021). *K. pneumoniae* O2 is the most heterogenous serotype characterized by the presence of many partial antigens (**Figures 2A, G**), among which O2ab was detected in the majority of O1 and O2ab clinical isolates tested (Whitfield et al., 1992; Trautmann et al., 1997; Trautman et al., 2004). In the *Proteus* spp. classification scheme the most heterogenous serogroup containing *P. mirabilis* strains is O23 with two subgroups (O23a,b,c; O23a,b,d) and with two OPSs structural variants among O23a,b,c representatives (Knirel et al., 2011).

The *Proteus* spp. classification scheme is based on the structural and serological diversity of LPS OPSs (Knirel et al., 2011). In the *Klebsiella* spp. classification scheme the crucial role is played by the heat-stable K-antigens, which are more diverse (77 serotypes) than the OPS parts (Podschun and Ullmann, 1998). However, the huge number of K serotypes and serological cross-reactions among them limit the K serotyping (Podschun and Ullmann, 1998; Brisse et al., 2006).

##### 3.2.1.2 Capsular polysaccharide

Hydrophilic polysaccharide capsules (K antigens) are the first virulence factors described for the genus *Klebsiella* and they are the most thoroughly studied ones (Brisse et al., 2006). The capsule surrounds the entire cell protecting it from drying out, phagocytosis and killing by serum and is associated with the development of the later stages of complex biofilm. Capsules are acidic structures, built of repeating units with four to six sugars and produced by the majority of uropathogenic *K. pneumoniae* strains (Schembri et al., 2005; Brisse et al., 2006). The size of *Klebsiella* CPSs influences the bacterial virulence. In contrast to highly encapsulated *K. pneumoniae* KP1-O which has been found to be extremely virulent in the murine burn wound sepsis model, the strain KP1-T with a smaller capsule (approximately one-third of that KP1-O) appeared to be comparatively nonvirulent (Cryz et al., 1984). The virulence of *K. pneumoniae* strains is also associated with a CPS structure. In less virulent K7 and K21a capsular antigens, the repetitive sequence of mannose-α-2/3-mannose is recognized by mannose-α-2/3-mannose-specific lectin of macrophages, which leads to lectinophagocytosis. On the other hand, the lack of mannose-2/3-mannose structures in more virulent K2 strains protects them against this process (Brisse et al., 2006). The capsule is so important in *K. pneumoniae* virulence because it may also influence the other virulence factors activity. It has been shown on *K. pneumoniae* strain C105 and its non-capsulated derivative that capsule expression results in impeding biofilm formation on the abiotic surface and agglutination of yeast cells, both processes mediated by type 1 fimbriae. This phenomenon has been suggested to result from the direct physical interference between type 1 fimbriae and the capsule (Schembri et al., 2005). CPS has been found to be a dominating (63%) component of the extracellular toxic complex (ETC), which is also composed of LPS (30%) and protein (7%). It has been shown that pure ETC of *K. pneumoniae* K8 strain exerts the cytotoxic effect on the rat embryo fibroblast cell line and changes the cell morphology. Although LPS accounts for ECT toxicity, the ETC effectiveness depends on the presence of a sufficient cell-associated capsule protecting the cell from phagocytosis (Al-Jumaily et al., 2012).

Among 78 K serotypes, 25 are mainly found in *Klebsiella* spp. clinical isolates causing bacteremia and the K2 serotype is predominant, followed by K1, in all *Klebsiella* spp. clinical isolates causing UTI, pneumonia and bacteremia (Podschun and Ullmann, 1998; Paczosa and Mecsas, 2016). Also, the K2 serotype, together with K1, is common among hvKP strains causing CA-PLA (community-acquired pyogenic liver abscesses). However, hypervirulence of K2 hvKP strains is believed to be connected with an increased expression of capsular material (hypermucoviscous phenotype) associated with the *rmpA* gene (a regulator of the mucoid phenotype) rather than the capsule serotype (Shon et al., 2013). Hypercapsulation increases resistance to complement killing or human neutrophil protein 1 and lactoferrin. The prevalence of more virulent K1 and K2 phenotypes among clinical *K. pneumonia* isolates results from their better capacity of survival in tissues and a higher resistance to lectinophagocytosis (Paczosa and Mecsas, 2016). In contrast to clinical isolates, the K33 and K69 serotypes dominated among environmental strains (*e.g.* from surface waters) (Podschun et al., 2001). Recently, on the basis of CPS locus and K-locus (KL) arrangement, series KL101-KL149, KL151, KL153-KL155 and KL157-159 have been additionally discovered, however their sugar composition remains unknown (Patro and Rathinavelan, 2019).

In *Proteus* spp. only four CPS structures have been described (two for *P. mirabilis* and two for *P. vulgaris* strains) (Beynon et al., 1992; Perry and MacLean, 1994; Rahman et al., 1997; Rahman et al., 1999). *Proteus* spp. CPSs consist of three to four saccharide residues and, similarly to *Klebsiella* CPSs, are acidic (due to the presence of uronosyl residues *e.g.* β-D-GalA in *P. mirabilis* WT19 or α-D-GlcA in *P. mirabilis* ATCC 49565). The acidic CPS is believed to act as a lubricant facilitating the migration of *Proteus* spp. swarmer cells (Rahman et al., 1997). However, in the *Proteus* spp. serological classification scheme, CPS is not taken into account due to the discovery that the CPSs of the two tested *P. mirabilis* 49565 and *P. vulgaris* ATCC 49990 strains had the same structures as their LPSs O chains (Beynon et al., 1992; Perry and MacLean, 1994).

What is interesting, while analyzing the selected *Klebsiella* spp. CPSs and *Proteus* spp. OPSs structures, common fragments could be observed as shown in the frames (**Figure 3**) (Lindberg et al., 1975; Dutton et al., 1978; Joseleau et al., 1978; Joseleau and Marais, 1979; Joseleau and Marais, 1988; Jansson et al., 1994; Erbing et al., 1995; Knirel et al., 2011; Kubler-Kielb et al., 2013). The first fragment (**Figure 3A**) is rather a common component of *K. pneumoniae* CPSs and it may be substituted by the lateral residue of β-D-GalA like in K8 (reference strain 1015) or K59 serotypes (Lindberg et al., 1975; Erbing et al., 1995). The mentioned K serotypes share the fragment, 3)- β-D-Glc*p*-(1→3)-β-D-Gal*p*(1-, with two *P. mirabilis* O serotypes, however this structure was not found in OPS of other known O serotypes of the *Enterobacterales* LPSs (Knirel, 2011). The components of fragment 3a are also present in the LPS OPS of *Providencia alcalifaciens* O9, O23, *Hafnia alvei* or *Edwardsiella ictaluri* MT 104 but in a different sequence and conformation, which makes the fragment unique for *P. mirabilis* OPS and *K. pneumoniae* CPS. The more common fragments of polysaccharides from many different bacterial species share more cross-reactions between specific antibodies and common epitopes can be observed in serological studies (Palusiak, 2021). The α-L-Rha residues are common components of *K. pneumoniae* CPSs (**Figures 3B, C, E**) which are rarely found in *Proteus* OPSs (*Proteus* spp. O22, O32 or O75) (**Figure 2P**, **Figure 3E**) (Joseleau and Marais, 1988; Athamna et al., 1991; Knirel et al., 2011; Kubler-Kielb et al., 2013). The different rhamnose disaccharides are also commonly found in OPSs of other *Enterobacterales* LPSs like: *Hafnia alvei*, *Citrobacter youngae*, *C. braakii*, *Escherichia hermannii* (Knirel, 2011). The fragment 2)-α-L-Rha-(1→2)-α-L-Rha(1-, depicted in **Figure 3E** as common for appropriate *K. pneumoniae* and *Proteus mirabilis* serotypes, is located in *H. alvei* 1222 OPS, where it is phosphorylated, in many *Shigella flexneri* O serotypes (Knirel, 2011).

The knowledge on polysaccharide fragments common to such a wide range of *Enterobacterales* would be helpful in obtaining broadly cross-reactive antibodies that would be also protective and, when given to patients, would neutralize LPS and prevent from septic shock development (Poxton, 1995). Another example of applying common polysaccharide fragments is ELISA construction where the fragments would be used as coated antigens recognized by specific antibodies from the patients sera. Such an assay, EndoCAB™, was applied for screening blood donors for high levels of cross-reactive antibodies specific to LPS core regions of *K. pneumoniae*, *E. coli*, *S. minnesota* and *Pseudomonas aeruginosa* R-mutants used as antigens (Poxton, 1995).

Sharing common fragments of polysaccharides by representatives of different bacterial genera is meaningful in the development of a multivalent vaccine with a broad spectrum of cross-protection *in vivo*. Such a vaccine may contain whole bacterial cells (inactivated, attenuated or cell lysates), the whole antigens or their fragments (epitopes) (Oloomi et al., 2020).

## 4 Vaccine formula based on *K. pneumoniae* and *P. mirabilis* antigens

There are three commercially available vaccines composed of inactivated enterobacterial strains, including *K. pneumoniae* and *Proteus* spp.: Uromune^®^ (sublingual spray), Urovac^®^ (administered *via* vaginal suppositories) and Urostim^®^ (administered orally), whose administration to volunteers led to a reduction of recurrent UTIs with slight potential adverse reactions (Nenkov, 2000; Uehling et al., 2003; Hopkins et al., 2007; Yang and Foley, 2018). However, applying a whole cell vaccine may be associated with some disadvantages like the non-specific immune responses and toxicity of such formulations (Oloomi et al., 2020; Assoni et al., 2021). The subunit of *K. pneumoniae* vaccines containing CPSs as antigens was tested in clinical trials, which showed encouraging response to the vaccine formula (Campbell et al., 1996). It is worth remembering that immune responses elicited by an isolated antigen are not long-lasting (lack of immunological memory) and are not T cell-dependent, thus antigen conjugation to carrier protein could enhance its immunogenicity (Lin et al., 2022). Lin et al. (2022) developed a new method of obtaining conjugated vaccine antigens by using K1 and K2 CPS depolymerases to receive depolymerized CPS without losing their modifications (related to CPS immunogenicity), which typically appear after applying standard chemical reagents. Vaccination of mice with K1 and K2 oligosaccharides conjugated with CRM197 carrier protein induced anti-CPS antibodies and protected mice from subsequent infection by the respective *K. pneumoniae* K-serogroup (Lin et al., 2022). The last type of the above-mentioned vaccines is the epitope-driven formula. It would be ideal if such a vaccine included many epitopes common to antigens of different bacterial species. The fragments (marked with frames in **Figure 3**) common for *K. pneumoniae* CPS and *P. mirabilis* OPS may be potential candidates for the multi-epitope formula development. Importantly, some of the fragments also occur in OPS of other *Enterobacterales* LPSs (Knirel, 2011), which gives a chance to obtain a broad spectrum of immune system inducers. It should be remembered that the construction of a vaccine of that type requires applying complex modern methodology *e.g.* bioinformatics tools for searching databases, multi-epitopes synthesis, cloning and protein expression methods. A novel multiepitope candidate vaccine based on the epitopes of nine common *E. coli* protein antigens, which were conserved for the epitopes of *K. pneumoniae* and *P. mirabilis* antigens and were MHC I and MHC II inducers, provided a significant protection in the bladder and kidneys in the UTI mice model (Oloomi et al., 2020).

Apart from the studies on the multiepitope vaccine including epitopes/fragments shared by both *K. pneumoniae* and *P. mirabilis* antigens, other investigations also concerned developing a vaccine formula based on one kind of antigen e.g. *Klebsiella* spp. capsule polysaccharide (mentioned above) or MR/K or *P. mirabilis* MrpH or Pta (Campbell et al., 1996; Lavender et al., 2005; Wang et al., 2016; Armbruster et al., 2018; Assoni et al., 2021).

MR/K-HA (type-3) - mannose-resistant *Klebsiella*-like hemagglutinins (19.5-21.5 kDa) agglutinate tannin treated ox erythrocytes and mediate binding of bacteria to human endothelial and uroepithelial cells, tubular basement membranes and Bowman’s capsules of the kidneys and urinary catheters (Podschun and Ullmann, 1998; Di Martino et al., 2003; Brisse et al., 2006; Sękowska and Gospodarek, 2007; Różalski et al., 2012). It has been showed that type 3 fimbrial shaft (MrkA) facilitates bacterial interactions and biofilm formation on abiotic surfaces including catheters, which may lead to CAUTIs development (Wu and Li, 2015; Langstraat et al., 2001; Schroll et al., 2010). MR/K-HA were first discovered in *Klebsiella* spp. strains and they are the main adhesive factors of *K. pneumoniae*, occurring more frequently in clinical than in sewage isolates (Di Martino et al., 2003; Brisse et al., 2006). Mice immunization with recombinant MrkA or purified type III fimbriae induced protection in a pneumonia model (Lavender et al., 2005; Wang et al., 2016; Assoni et al., 2021).

MR/P fimbriae - mannose resistant *Proteus* like fimbriae (agglutinating untreated erythrocytes in the presence of mannose) are the most important fimbriae for enhancing the formation of *P. mirabilis* initial biofilm, involved in the upper urinary tract colonization (especially the bladder), pyelonephritis development and showing autoaggregative properties (Jansen et al., 2004). Administration of the purified *P. mirabilis* HI4320 MR/P fimbriae to mice by different routes provided protection against reinfection with a homologous strain in 63% of animals in the case of intranasal or transurethral routes (Li et al., 2004; Armbruster et al., 2018). When purified recombinant MrpA (the main structural subunit of MR/P) was tested as a vaccine antigen in an ascending and a hematogenous model of UTI in mice, the subcutaneously immunized animals appeared to be protected against *P. mirabilis* UTI (Pellegrino et al., 2003).

*Proteus* toxic agglutinin (Pta) is a surface associated calcium-dependent subtilisin-like alkaline serine protease. Pta exhibits bifunctional action by mediating the autoaggregation of bacterial cells or eliciting cytopathic effects on cultured kidney and bladder epithelial cells. Pta seems to be pathogen specific and its activity during infection is probably urease dependent. The passenger domain containing catalytic residues (Ser366, His147, Asp533) accounts for Pta cytotoxic activity (Alamuri and Mobley, 2008). Pta is a promising vaccine antigen - mice immunization with purified intact Pta or the passenger domain (Pta-α), each conjugated with cholera toxin (CT) protects significantly against *P. mirabilis* UTI, mainly in the upper urinary tract (Alamuri et al., 2009).

## 5 *Klebsiella* spp. and *Proteus* spp. LPSs in the serological studies

Structural similarities between polysaccharides of *Klebsiella* spp. and *Proteus* spp. strains were revealed in numerous cross reactions of *Klebsiella* LPSs with polyclonal rabbit sera specific to 11 *Proteus* spp. strains (Palusiak, 2015; Palusiak, 2021). Using the Western blotting technique allowed showing the reactions which mainly concerned the high-molecular-species containing LPS moieties with O-polysaccharide chains. The patterns typical for LPS low-molecular-mass-species corresponding to core-lipid A fractions were also noticed in the case of four *Klebsiella* LPSs and *P. penneri* 2 antiserum. Considering the fact that the LPS core regions of the representatives of both genera are very similar to each other, mainly in their inner parts, the cross reactions concerning this region should be more common (Palusiak, 2021). However, it ought to be remembered that the polysaccharide part of LPS may restrict an access to the core region and thus the reactions with core-specific-antibodies may not have been detected (Palusiak, 2013).

The reactions differed in their intensity depending on the used serum and they were noticed not only for the representatives of different *Proteus* O serogroups but also for different species of the genus *e.g*. *P. mirabilis*, *P. vulgaris* or *P. penneri*. This observation might be crucial for the future selection of vaccine antigens which should be characterized by having common fragments with as many LPSs as it is possible to induce the broad spectrum of protection. It should be remembered that LPS exhibits high toxicity which limits the possibilities of using this antigen in the vaccine formula (Assoni et al., 2021). However polysaccharides only induced T-cell-independent response, thus a conjugated vaccine would be more recommended as promoting strong, long lasting responses in all age groups of individuals (Assoni et al., 2021; Lin et al., 2022).

An important question which should be addressed is whether the cross-reactions appear in opposite systems *e.g. Proteus* LPSs and *Klebsiella* antisera and *Klebsiella* LPSs and *Proteus* antisera. In the mentioned studies such cross-reactions appeared in the case of four LPSs, *P. penneri* 19, 22 and 60 and *K. oxytoca* 0.062, which may indicate them as potential vaccine antigens. However, the research should be further completed with the data from detailed structural studies to show which O antigen fragments contribute to cross-reactions (Palusiak, 2021). So far, antigen O of *Shigella sonnei* has been successfully used in vaccines administered to humans in clinical studies (Launay et al., 2017). Long-termed catheterized patients and those prone to the recurrent UTIs of *Klebsiella* spp. and *P. mirabilis* etiology may be predestined to receive the LPS vaccine *via* the intranasal route providing the highest protection (Armbruster et al., 2018).

Summarizing, both *K. pneumoniae* and *P. mirabilis* are important etiological factors of nosocomial infections, especially those affecting the urinary tract, which may be infected by both pathogens simultaneously (Wu and Li, 2015; O’Hara et al., 2000; Brisse et al., 2006; Macleod and Stickler, 2007; Różalski et al., 2012). Such polymicrobial infections are often difficult to treat due to the development of biofilm, in which the cells of both species may cooperate in reaching the upper parts of the tract, and to frequent multidrug resistance of the bacteria (Macleod and Stickler, 2007; Jacobsen et al., 2008; Cuevas et al., 2010; Drzewiecka and Lewandowska, 2016). Thus, there is a need to acquire better knowledge on the virulence factors of both pathogens, especially those exhibiting common features. For instance, the examination of common fragments in highly immunogenic polysaccharides *i.e. K. pneumoniae* CPSs and *P. mirabilis* OPSs (**Figure 3**) and detection of various cross-reactions (Palusiak, 2015; Palusiak, 2021) are of high importance for the creation of a vaccine protecting against both pathogens.

## Author contributions

AP conceived and designed of the review and prepared the whole manuscript.

## Conflict of interest

The author declares that the research was conducted in the absence of any commercial or financial relationships that could be construed as a potential conflict of interest.

## Publisher’s note

All claims expressed in this article are solely those of the authors and do not necessarily represent those of their affiliated organizations, or those of the publisher, the editors and the reviewers. Any product that may be evaluated in this article, or claim that may be made by its manufacturer, is not guaranteed or endorsed by the publisher.

## Glossary

**Table:**

3-OH-C_14_	3-hydroxytetradecanoic acid
L-Ara*p*4N	4-amino-4-deoxy-L-arabinopyranose
AS	ankylosing spondylitis
ATCC	American Type Culture Collection
BSIs	bloodstream infections
CAP	community-acquired pneumonia
CA-PLA	community-acquired pyogenic liver abscess
CAUTI	catheter-associated UTIs
Cho*P*	choline phosphate
ETC	extracellular toxic complex
Etn*P*	ethanolamine phosphate
CPS	capsular polysaccharide
Fuc	fucose
D-Fuc3N	3-amino-3-deoxy-D-fucose
Gal*p*	galactopyranose
Gal*f*	galactofuranose
GalA	galacturonic acid
GalAN	GalA amidated by aliphatic polyamines
GalN	galactosamine
D-GalNAc	2-acetamido-2-deoxy-D-galactose
Gal*o*NAc	open-chain of GalNAc
Glc	glucose
GlcA	glucuronic acid
GlcN	glucosamine
D-GlcNAc	2-acetamido-2-deoxy-D-glucose
D-Gro-1-*P*	D-glycerol 1-phosphate
HAPS	hospital-acquired pneumonia
LD-Hep	L-*glycero*-D-*manno*-heptose
DD-Hep	D-*glycero*-D-*manno*-heptose
hvKP	hypervirulent *K. pneumonia*
Kdo	3-deoxy-D-*manno*-oct-2-ulosonic acid
LPS	lipopolysaccharide
LPB	LPS binding protein
L-Lys	L-lysine
Man	mannose
Me	methyl group
MR/K-HA	mannose-resistant *Klebsiella*-like hemagglutinin
MR/P	mannose resistant *Proteus* like fimbriae
*Pyr*	*pyruvic acid*	
*R*-3HOBu	(*R*)-3-hydroxybutanoic acid
*R*-Cet-Etn*P*	*N*-[(*R*)-1-carboxyethyl]ethanolamine phosphate
RA	rheumatoid arthritis
Rha	ramnose
Rib*f*	ribofuranose
*rmpA*	regulator of the mucoid phenotype
OPS	O-polysaccharide
OS	oligosaccharide
*P*Etn	2-aminoethyl phosphate
Pta	*Proteus* toxic agglutinin
L-Ser	L-serine
S	smooth
SR	semi-rough
Suc	succinic acid
Sug	4-deoxy-*threo*-hex-4-enopyranosyluronic acid group
UTIs	urinary tract infections
TLR	Toll-like receptor
T4SS	type IV secretion system
Qui4N	4-amino-4-deoxy-D-quinovose
